# Transcriptome Profile Analysis Identifies Candidate Genes for the Melanin Pigmentation of Skin in Tengchong Snow Chickens

**DOI:** 10.3390/vetsci10050341

**Published:** 2023-05-11

**Authors:** Xiannian Zi, Xuehai Ge, Yixuan Zhu, Yong Liu, Dawei Sun, Zijian Li, Mengqian Liu, Zhengrong You, Bo Wang, Jiajia Kang, Tengfei Dou, Changrong Ge, Kun Wang

**Affiliations:** 1 College of Animal Science and Technology, Yunnan Agricultural University, Kunming 650201, China; zxnzyx1119@163.com (X.Z.); zhuyxyn@126.com (Y.Z.); lydzq05091025@163.com (Y.L.); lzj01071217@163.com (Z.L.); lmq7498a123@163.com (M.L.); 13888796612@163.com (B.W.); kjj730248@163.com (J.K.); tengfeidou@sina.com (T.D.); 2 Shenzhen Hualong Sunda Information Technology Co., Ltd., Shenzhen 518000, China; lovexiaji@163.com; 3 Yunnan Animal Science and Veterinary Institute, Kunming 650224, China; sdw17787273236@163.com; 4 Zhaotong Animal Husbandry and Veterinary Technology Extension Station, Zhaotong 657000, China; youzhr1968@163.com

**Keywords:** Tengchong Snow chicken, skin, luminance value (L value), melanin, transcriptome, candidate genes

## Abstract

**Simple Summary:**

Chicken meat with high melanin content in black-boned chickens is considered a highly nutritious food with potential medicinal value. Tengchong Snow chicken is one of the valuable poultry resources in Yunnan Province, and this chicken is usually dominated by dark meat. However, during the rearing process, we found that a small number of white meat traits still existed in this chicken population. The purpose of this study was to investigate the deposition pattern and molecular mechanism of melanin formation in Tengchong Snow chickens. We selected Tengchong Snow black meat chicken (Bc) and white meat chicken (Wc) as the study subjects to determine differences in the luminance value (L value) and melanin content in their skin tissues at 1, 42 and 90 days; the L value and melanin content of the skin were measured using color chromatography, ELISA kits, and enzyme markers. We found that age had an effect on skin L values and melanin content. The L value gradually increased with age, while melanin content showed the opposite trend. Moreover, the L value of the skin tissue was negatively correlated with melanin content. Finally, we identified the *TYR*, *DCT* and *EDNRB2* genes as being the possible main effector genes affecting skin pigmentation using transcriptome profiling; the expression of the *TYR*, *DCT*, *MC1R*, *EDNRB2*, *GPR143*, *MITF*, and *TYRP1* genes also gradually decreased with increasing day age. In conclusion, this study describes and reveals the differences in L values and melanin content in chicken skin tissues. The results of the study provide a valuable theoretical basis for future breeding programs of Tengchong Snow chickens, as well as a foundation for studies on skin tone deposition and a theoretical reference for the future conservation and utilization of black-boned chickens.

**Abstract:**

Tengchong Snow chickens are one of the most precious, black-boned chickens in Yunnan province and usually produce black meat. However, we found a small number of white meat traits in the chicken population during feeding. In order to determine the pattern of melanin deposition and the molecular mechanism of formation in the Tengchong Snow chicken, we measured the luminance value (L value) and melanin content in the skin of black meat chickens (Bc) and white meat chickens (Wc) using a color colorimeter, ELISA kit, and enzyme marker. The results showed that the L value of skin tissues in black meat chickens was significantly lower than that of white meat chickens, and the L value of skin tissues gradually increased with an increase in age. The melanin content of skin tissues in black meat chickens was higher than that of white meat chickens, and melanin content in the skin tissues gradually decreased with an increase in age, but this difference was not significant (*p* > 0.05); the L value of skin tissues in black meat chickens was negatively correlated with melanin content, and the correlation coefficient was mostly above −0.6. In addition, based on the phenotypic results, we chose to perform the comparative transcriptome profiling of skin tissues at 90 days of age. We screened a total of 44 differential genes, of which 32 were upregulated and 12 were downregulated. These DEGs were mainly involved in melanogenesis, tyrosine metabolism and RNA transport. We identified *TYR*, *DCT*, and *EDNRB2* as possible master effector genes for skin pigmentation in Tengchong Snow black meat chickens through DEGs analysis. Finally, we measured the mRNA of *TYR*, *DCT*, *MC1R*, *EDNRB2*, *GPR143*, *MITF*, and *TYRP1* genes through a quantitative real-time polymerase chain reaction (qPCR) and found that the mRNA of all the above seven genes decreased with increasing age. In conclusion, our study initially constructed an evaluation system for the black-boned traits of Tengchong Snow chickens and found key candidate genes regulating melanin deposition, which could provide an important theoretical basis for the selection and breeding of black-boned chickens.

## 1. Introduction

As a valuable genetic resource of domestic chickens in oriental countries, the black-boned chicken is an important animal model for the study of melanin deposition and is an extremely popular meat product in the consumer market [1]. This is not only due to its tasty meat but, more importantly, its tissues, such as skin, muscle, and internal organs, which are rich in melanin [2]. The skin color of chickens, as one of the important economic traits in the production process, is an important research direction for many breeders in breed (matching lines) selection, promotion and commercial packaging [3]. Studies have shown that the black-boned chicken has a unique impact on human health, and the deeper its skin color is, the more profound its impact on health, and it can now be used as a medical health chicken breed due to the effects of melanin in scavenging free radicals, anti-oxidation, delaying aging, and improving the immunity of the body [4,5]. The skin color of black-boned chickens has thus become one of the important influencing factors for consumers when deciding whether to purchase them. However, the lack of attention and concern in farmers about the whitening or lightening of the skin color of black-boned chickens during the breeding process has slowed down the breeding selection process, which, in turn, has had a significant impact on the economic income of farmers [6].

Melanin, a natural pigment, can be divided into two main types: eumelanin and phaeomelanosome [7]. Its synthesis and distribution are influenced by many genes, transcription factors, proteins, and signaling pathways, making melanin deposition patterns in the animal organism diverse. Dou et al. [8] used transcriptome analysis to identify 25 differentially expressed genes and 11 transcription factors in the melanogenesis pathway and found that *TFAP2B*, *TFAP2A*, *TCF21*, and *ELF3* genes may be the key genes regulating tyrosine metabolism and melanogenesis in chicken muscle. Zhang et al. [9] identified 649 differentially expressed genes on the black and white skin of Xianyang chickens and found *KIT*, *ASIP*, *TYR*, and *OCA2* genes to be the main regulatory genes for melanin deposition in the skin of Xianyang chickens. Zhang et al. [10] revealed through high throughput sequencing that lncRNA LMEP on the regulatory mechanism of melanin deposition in the skin of Xichuan chickens. The deposition and expression of melanin content and pigment-related genes could vary widely across breeds, sexes, ages, and tissues. The study of Khumpeerawat et al. [11] found that chicken breed, sex, and age could have an effect on the melanin content and expression of related genes. Jian et al. [12] studied the luminance values, melanin content, and mRNA expression levels in pigment-related genes such as *ASIP*, *MC1R*, *MITF*, *TYR*, and *TYRP1* in different chicken breeds and tissues using colorimetry, enzymatic digestion, and qPCR techniques.

The Tengchong Snow chicken, one of the most excellent local chicken breeds in Yunnan province, is mainly produced in the western foothills of Gaoligong mountain, where snow accumulates in winter, and is named because of its white feathers. In a previous study, we found that the Tengchong Snow chicken population not only has black meat, but also white meat, with relatively better meat quality, tenderness, taste, and freshness compared to local chicken breeds or commercial broilers [13]. Currently, the molecular mechanism of melanin formation and deposition in the skin of Tengchong Snow chickens is still unclear. Therefore, in order to determine the regularity of melanin production and deposition in the skin tissue of Tengchong Snow chickens and the screen-related candidate genes affecting melanin deposition, our study aimed to explore the L value and melanin content of the skin in Tengchong Snow chickens at different growth stages, and explore the related candidate genes of melanin deposition so as to reveal the molecular mechanism of melanin production and deposition in its skin. These results can provide an important theoretical basis for the breeding of black-boned characteristics in the Tengchong Snow chicken.

## 2. Materials and Methods

### 2.1. Animal Experimentation Ethical Statement

All procedures conducted with the chickens were approved by the Yunnan Agricultural University Animal Care and Use Committee (approval ID: YAUACU C01). Animal use and care were in accordance with the guidelines for the care and utility of experimental animals prescribed by the Chinese Ministry of Agriculture and Guide for the Care and Use of Laboratory Animals and published by the England National Research Council.

### 2.2. Chicken, Diet, and Housing

A total of 300 healthy Tengchong Snow chickens (150 each of the black chicken meat and white chicken meat) were selected for this experiment, of which the black chicken meat of Tengchong Snow chickens was the experimental group, and the white meat chickens formed the control group. At the 1st, 42nd, and 90th days of age, 10 black meat and 10 white meat Tengchong Snow chickens were randomly selected for slaughter, and their feed and water supply were stopped 12 h before slaughter. When slaughtering, the animals were killed by bleeding from the neck. After depilation, the skin tissues (with the surface fat and fascia removed) were sampled and put into a cryopreservation tube. After being frozen by liquid nitrogen, the samples were stored in a freezer at −80 °C for RNA extraction. In addition, skin tissues from the same part were collected and stored in a freezer at −20 °C for the determination of melanin content.

### 2.3. Determination of Luminance Values and Melanin Content

The luminance value (L value) on the skin of each experimental chicken was measured using a color colorimeter. All L values were obtained from two areas of each tissue, and the higher the reading of the L value, the lower the black color, the less melanin deposition, and vice versa. A whiteboard calibration was performed before the measurement, and the colorimeter was in close contact with the muscle and skin during the measurement to prevent measurement errors caused by light leakage.

The phaeomelanosome, eumelanin, and total melanin contents in the skin tissue of two different meat colors in Tengchong Snow chickens at different ages were determined using ELISA kits.

### 2.4. Extraction of Total RNA

The total RNA extraction kit (Bao Bioengineering Co., Ltd., Dalian, China) was used to extract the total RNA from the skin tissues of Tengchong Snow chickens at the age of 90 days, according to the requirements of the instructions. Purity was detected by the Nanodrop 2000 spectrophotometer (Nanodrop, Wilmington, DE, USA). The OD260/OD280 ratio of all samples was 1.8 to 2.0. The concentration of the total RNA was determined using the Qubit 2.0 Fluorometer (Thermo Fisher Scientific Inc., Waltham, MA, USA), and integrity was detected using the Agilent 2100 Bioanalyzer (Agilent Technologies, Inc., Santa Clara, CA, USA). The total RNA samples were stored at −80 °C until further use. Six replicates were prepared for each of the black and white meat chickens for transcriptome sequencing.

### 2.5. Construction and Sequencing of RNA-Seq Libraries

Firstly, eukaryotic mRNA was enriched with oligo(dT) magnetic beads, rRNA was removed, and prokaryotic mRNA was enriched with the Ribo-ZeroTM magnetic kit (Epicentre, Madison, WI, USA). Subsequently, to enrich mRNA, two rounds of hybridization to oligo (dT) beads were performed on a 7 μg total RNA of each sample, and the enriched mRNA was then broken into short fragments using a break buffer before being reverse-transcribed into cDNA using random primers. The second strand, cDNA, was synthesized using DNA polymerase I, RNase H, dNTP, and a buffer. dNTP and the buffer were used to synthesize the second strand of cDNA. cDNA fragments were then purified using the QIAquick PCR extraction kit (Canspec Scientific Instruments Co., Ltd., Shanghai, China), end-repaired, and poly-A tails were added and ligated to Illumina sequencing adapters. Finally, the ligated products were size-selected by agarose gel electrophoresis, PCR amplified, and sequenced using Illumina HiSeq2500 (Kidio Biotechnology Co., Ltd., Guangzhou, China).

### 2.6. RNA-Seq Data Acquisition, Quality Control, and Processing Raw Data Quantification

RNA-seq data were filtered by fastp software (version 0.18.0) to remove reads containing >10% of unknown nucleotides from the adapters and >50% of the low-quality (Q value ≤20) base. Reads were removed after being compared to ribosomal RNA (rRNA) and the reference genome using the short read alignment tools Bowtie2 (version 2.2.8) and HISAT2.X (version 2.4) after performing the comparison. Clean mapping reads were assembled for each sample in a reference-based manner using StringTie v1.3.1, respectively, to quantify gene abundance. For each transcribed region, FPKM (fragments per kilobase of transcript per million mapped reads) values were calculated using StringTie software to quantify its expression abundance and variation.

RNA differential expression analysis was performed between the two different groups (and between two samples by edgeR) by DESeq2 software v1.40.1. The genes/transcripts with false discovery rate (FDR) parameters below 0.05 and an absolute fold change ≥2 were considered differentially expressed genes/transcripts. Correlation analysis was performed by R software, and the correlation of two parallel experiments was to assess the reliability of the experimental results as well as the stability of the operation.

### 2.7. Enrichment Analysis

The GO (Gene Ontology Consortium), KEGG (Kyoto Encyclopedia of Genes and Genomes), and Reactome database were used for the enrichment analysis of pathways of differentially expressed genes to investigate the possible biological functions.

### 2.8. Verification of the RNA-Seq Results

A qPCR test was performed using the Bio-Rad CFX96 real-time PCR platform (Bio-Rad Laboratories. Inc., Hercules, CA, USA) to validate 7 differentially expressed genes (DEGs) during tyrosine metabolism and melanogenesis, and primer information is provided in Table S1. The total RNA extracted from the skin tissues of Tengchong Snow chickens at the 1st, 42nd, and 90th days of age was analyzed using the same method as transcriptome analysis. qPCR analysis was performed using a PrimeScript^TM^ RT Reagent Kit and SYBRR Premix ExTaq II (Tli RNase H Plus; Takara, Dalian, China). The abundance of mRNA was analyzed using the 2^−ΔΔCt^ method, and ACTB was used as an internal control for the normalization of the results. Three replicates were set for each sample, and the mRNA expression was calculated by the measured average value. Differential gene expression-original data are provided in Table S2.

### 2.9. Statistical Analysis

Data statistics were processed and analyzed using SPSS 13.0 (SPSS, Chicago, IL, USA), Prism 9.0 (GraphPad Software, San Diego, CA, USA), and Excel 2016 (Microsoft Office, Washington, DC, USA). The significance of the variance test and correlation analysis were performed using independent sample t-tests and a correlation coefficient (Pearson method).

## 3. Results

### 3.1. Comparative Analysis of the Luminance Value and Melanin Content

**Figure 1:**
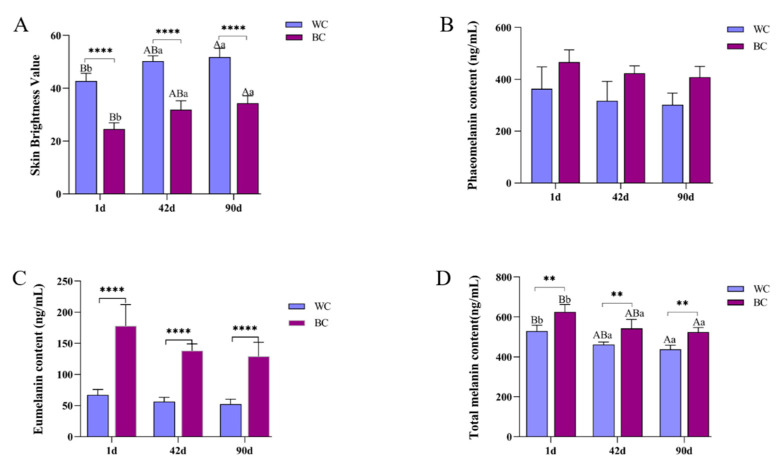
Comparative analysis of brightness values and melanin content in the skin of Tengchong Snow black meat and white meat chickens. (**A**) Significance analysis of skin luminance values in Tengchong Snow chickens. (**B**) Significance analysis of phaeomelanin content in the skin of Tengchong Snow chickens. (**C**) Significance analysis of eumelanin content in the skin of Tengchong Snow chickens. (**D**) Significance analysis of total melanin content in the skin of Tengchong Snow chickens. Note: “**” indicates a significant difference (*p* < 0.05); “****” indicates a highly significant difference; different lowercase letters indicate a significant difference (*p* < 0.05); different capital letters indicate highly significant differences (*p* < 0.01); the same letters or no letters on the shoulder indicate insignificant differences (*p* > 0.05).

In this study, the luminance values in skin tissues were evaluated using a color colorimeter. As can be seen from Figure 1A, the skin L values of black meat chickens and white meat chickens in Tengchong Snow chickens showed highly significant differences (*p* < 0.01) at the same age, and the L values of both black meat chickens were lower than those of white meat chickens. The L value in the skin tissue of black meat chickens and white meat chickens at different day ages gradually increased with the increase in day ages. From Figure 1B, the content of phaeomelanosome in the skin tissue of black meat chickens of the same age is higher than that of white meat chickens, but the difference is not significant. As shown in Figure 1C, the content of eumelanin in the skin tissue of black meat chickens was significantly higher than that of white meat chickens and gradually decreased with an increase in age. From Figure 1D, the content of the total melanin in the skin tissue of black meat chickens was higher than that of white meat chickens. At the same time, the total melanin, eumelanin, and phaeomelanosome in the skin tissues of Tengchong Snow black meat chickens and white meat chickens at different day ages decreased gradually with an increase in day ages.

### 3.2. Correlation Analysis between Luminance Value and Melanin Content

As shown in Table 1, the L values of the skin tissues in Tengchong Snow chickens were significantly negatively correlated with their total melanin, eumelanin, and phaeomelanosome contents (*p* < 0.05).

### 3.3. Skin Transcriptome Analysis: Differentially Expressed Genes and KEGG Enrichment Analysis

#### 3.3.1. Functional Enrichment Analysis of Differentially Expressed Genes

**Figure 2:**
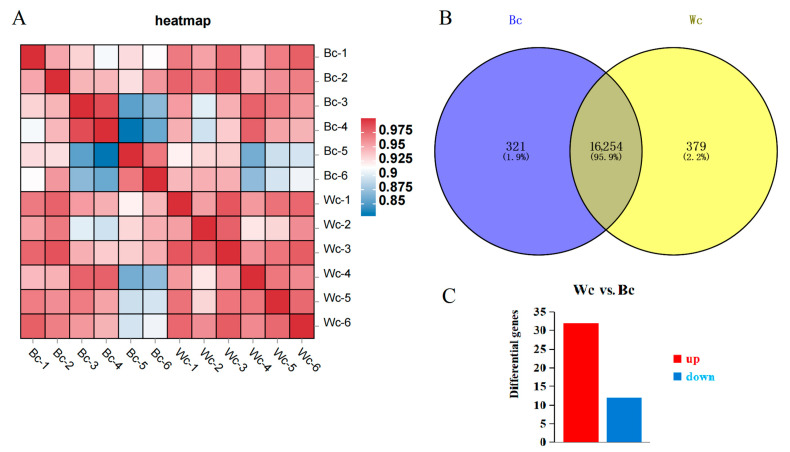
The differentially expressed genes that are unique or shared between Wc and Bc. (**A**) Sample of correlation analysis. (**B**) Overview of the number of differentially expressed genes (DEGs) in skin tissues. (**C**) Venn diagram showing Wc vs. Bc group-specific expression profiles.

A total of 79.58 G of raw data were obtained from RNA sequencing. From the comparison between each sample and the reference genome (Table S3), the comparison rate of black meat chicken was more than 88.16~93.75%, and the comparison rate of white meat chicken was between 87.55% and 93.57%, indicating that the utilization rate of the 2 groups of data was good and could meet the subsequent requirements. Among them, 73.18% were located in the exon region and 11.7% in the introns (Table S4). The average percentages of Q20 and Q30 bases were >97.14% and >92.57%, respectively (Table S5). After the quality control, the average cleaning reading of each sample was 52,793,503. The correlation analysis of gene expression level shows that the samples were highly correlated (Figure 2A), which indicates that the selection of experimental samples was reliable.

As shown in Figure 2B, 16,954 genes were expressed in the skin of black meat chickens and white meat chickens in Tengchong Snow chickens, of which 379 were specifically expressed in black meat chickens, 321 genes were expressed in white meat chickens, and 16,254 genes were expressed in the skin of both black meat and white meat chickens. A total of 44 differential genes were obtained from Wc vs. Bc, including 32 up-regulated genes and 12 down-regulated genes (Figure 2C).

**Figure 3:**
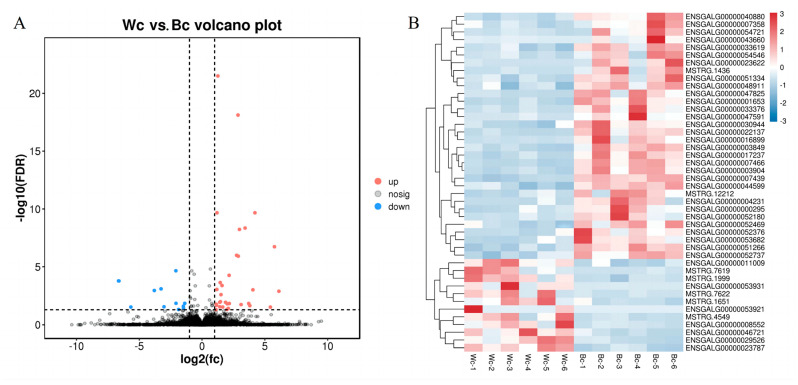
Differentially expressed genes of black and white meat chickens. (**A**) Volcano plot of Wc vs. Bc differentially expressed genes. (**B**) Heatmap of Wc vs. Bc differentially expressed genes.

The visualization of skin in differentially expressed genes is shown by a volcano plot. The volcano plot takes multiple gene expression differences as the *x* axis (log2 value of the differential multiple, positive value for the up-regulated, negative value for the down-regulated), and the *p* value of the difference as the *y* axis (−log10 value of *p* or FDR, both positive. The smaller the *p* or FDR value, that is, the stronger the statistical significance, the greater the −log10 value). As shown in Figure 3A, the volcano plot can clearly show the up-regulation and down-regulation of differentially expressed genes and their significance, with the red part indicating up-regulated differentially expressed genes, the blue part indicating down-regulated differentially expressed genes, and the blue part indicating genes with an insignificant differential expression. The closer the genes are to the two ends, the greater the degree of difference.

The distribution of differentially expressed genes in the skin of black meat and white meat chickens (Wc vs. Bc) in Tengchong Snow chickens in the samples was subjected to heatmap clustering analysis, and the results are shown in Figure 3B. It can be seen that the differentially expressed genes in the skin of the black meat chicken (Bc) in Tengchong Snow chickens showed an extremely consistent expression of abundance in each group, and were significantly different from the control group.

#### 3.3.2. GO Functional Enrichment Analysis

**Figure 4:**
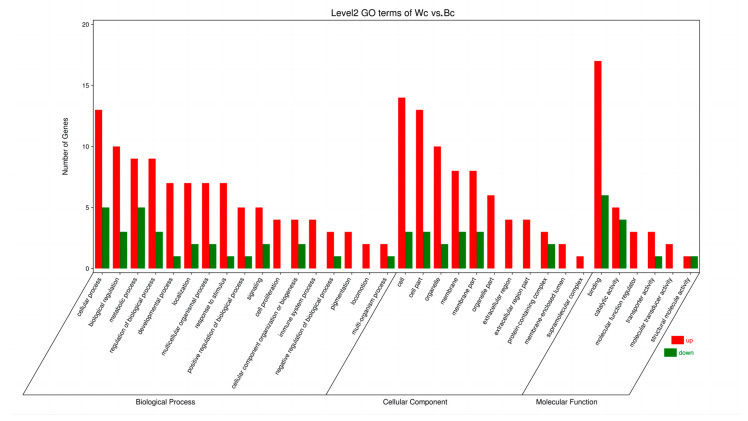
Significantly different GO items corresponding to different categories in the analyzed chicken skin. Note: red means up bar, green means down bar.

The GO function enrichment analysis of 44 differential genes on Wc vs. Bc showed that 35 significant items (*p* < 0.05) were enriched in their molecular function, biological process, and cell composition in the skin of Tengchong Snow chickens. From Figure 4, 11 significant items on cellular components, 6 significant items on molecular functions, and 18 significant items on biological processes can be observed.

#### 3.3.3. KEGG Pathway Analysis

**Figure 5:**
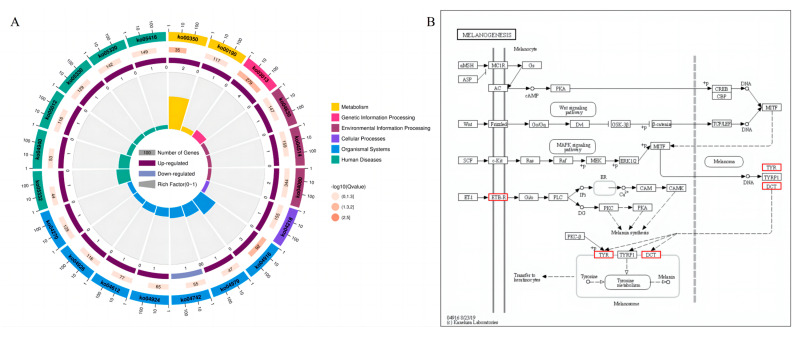
KEGG enrichment analysis of Wc vs. Bc. (**A**) Significantly enriched KEGG pathways. (**B**) The differentially expressed skin color genes identified in the analyzed chicken skin and their involvement in the melanogenesis pathway.

Figure 5A shows the top 20 pathways of KEGG analysis in skin tissue. At the threshold of a *p* value < 0.05, three significant KEGG pathways were generated on the skin, namely melanogenesis, tyrosine metabolism and RNA transport. At the same time, we paid more attention to the melanogenesis pathway in this study. As shown in Figure 5B, genes such as *EDNRB*, *TYR*, and *DCT* were mainly involved in the melanogenesis of the skin tissue in Tengchong Snow chickens.

#### 3.3.4. Reactome Enrichment Analysis

**Figure 6:**
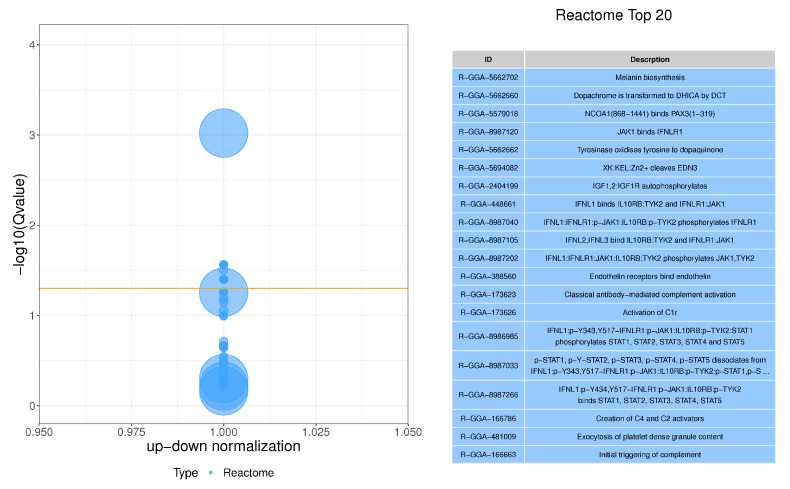
Reactome analysis of skin tissue DEGs. The first 20 Reactome pathways. The size of the dots in the figure represents Gene Count, and the orange line represents the threshold Q/*p*-value = 0.05, indicating that bubbles above this threshold line are significant.

The enrichment analysis of the Reactome pathway can provide us with a deeper understanding of biochemical, metabolic, and signal transduction pathways in differential genes. In this study, 26 significant pathways were found in the skin tissue of Tengchong Snow chickens, and Figure 6 shows the first 20 pathways, which mainly involved cell processes, environmental information processing, genetic information processing, human diseases, metabolism, and biological systems.

#### 3.3.5. Protein Interaction Network Analysis

**Figure 7:**
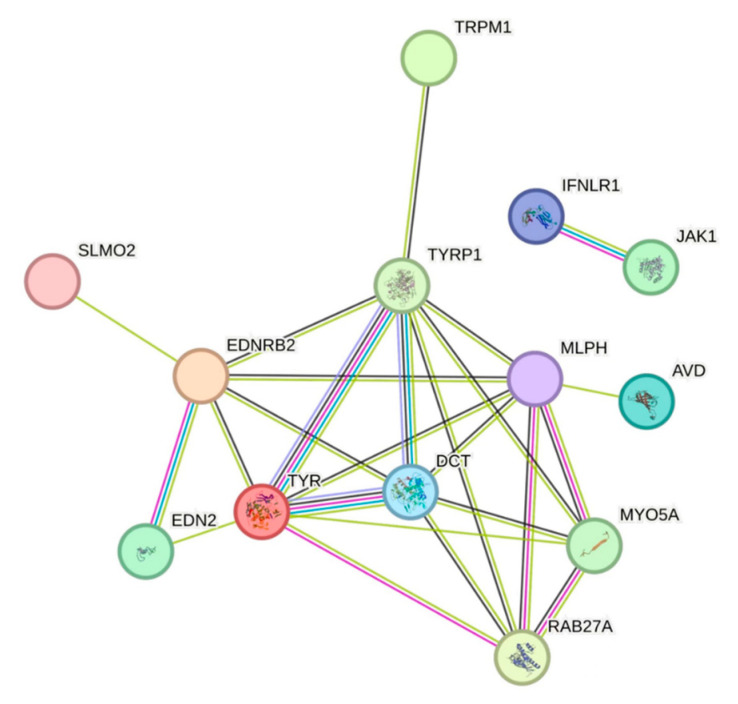
Protein network interactions map.

We mainly applied the interaction relationships in the STRING protein interaction database (http://string-db.org, accessed on 26 January 2022) for differential gene protein interaction network analysis (Figure 7). The selected intra-module genes were used for module visualization and were combined with the KEGG pathway enrichment analysis and protein interaction network analysis to identify *TYR*, *DCT*, *EDNRB2*, and *TYRP1* as the possible major regulatory genes affecting skin pigmentation in Tengchong Snow chickens.

### 3.4. Validation of Differentially Expressed mRNA in Chicken Skin

**Figure 8:**
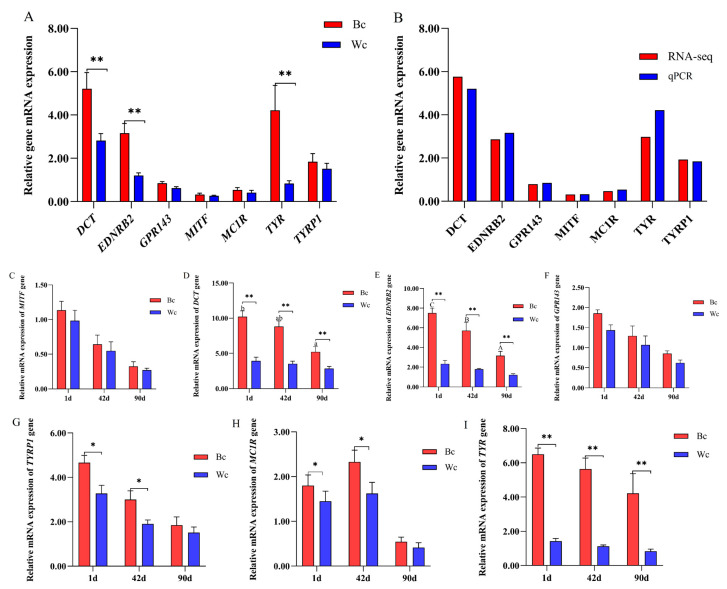
DEG was verified by qPCR analysis. (**A**) The results of qPCR in the skin tissue of Tengchong Snow chickens. (**B**) Comparison of transcriptome sequencing and qPCR results. (**C**) Relative mRNA expression of *MITF* gene. (**D**) Relative mRNA expression of *DCT* gene. (**E**) Relative mRNA expression of *EDNRB2* gene. (**F**) Relative mRNA expression of *GPR143* gene. (**G**) Relative mRNA expression of *TYRP1* gene. (**H**) Relative mRNA expression of *MC1R* gene. (**I**) Relative mRNA expression of *TYR* gene. “*” indicates a significant difference (*p* < 0.05); “**” indicates a highly significant difference (*p* < 0.01).

The results of transcriptome sequencing analysis showed that *TYR*, *DCT*, *MC1R*, *EDNRB2*, *GPR143*, *MITF*, and *TYRP1* were differentially expressed genes in Tengchong Snow chickens. To validate the transcriptome sequencing results, we examined the expression of *TYR*, *DCT*, *MC1R*, *EDNRB2*, *GPR143*, *MITF*, and *TYRP1* genes in the skin tissues of 90-day-old Tengchong Snow black and white meat chickens using qPCR technology. The qPCR results were consistent with the transcriptome sequencing results, indicating that the transcriptome sequencing results were reliable (Figure 8A,B). We also examined the expression of these 7 genes at 1 d and 42 d. As shown in Figure 8D,E,I, the expression of *EDNRB2*, *TYR*, and *DCT* genes was significantly different in the skin of both black and white meat Tengchong Snow chickens at the three-day-old stage. As shown in Figure 8C,F, there were no significant differences in the expression of *MITF* and *GPR143* genes in the skin tissue of Tengchong Snow black and white meat chickens at the three-day-old stage. The expression of *MC1R* and *TYRP1* genes was significantly different between the black and white meat chickens at 1 and 42 days of age, but no difference was detected at 90 days of age.

## 4. Discussion

The degree of black color in the skin of black-boned chickens, as a major selection target in the rearing process, is determined by the amount of melanin deposited, and consumers often make their purchase decisions based on the degree of black color in the chicken’s skin [14]. Therefore, some experts and scholars have attempted to classify the degree of black color of the black-boned chicken by visual classification. Kriangwanich et al. [15] described the muscle degree of black color in Thai black-bone chickens of different sexes through macroanatomical studies and found that the degree of black color did not differ between sexes but differed between specific skeletal muscles. Nganvongpanit et al. [16] studied the degree of black color in the study distribution of melanin in 32 organ tissues, including the brain, lung, muscle, spleen, and skin, and showed that melanin was distributed differently in different organs and did not differ significantly by sex. However, there were some subjective factors in assessing the degree of black color and lightness in black-bone chickens by visual classification, and several independent and experienced judges were needed to make a reliable evaluation, which could waste a lot of human and financial resources. It has been confirmed that the system of L*a*b* color parameters measured using a colorimeter is the most sensitive and reliable [17]. Wang et al. [14] used a colorimeter to measure skin color in the lateral thorax region of the cross progeny of Dongxiang blue eggshell chickens and Jiangshan black-bone chickens at different day-old stages, and the results showed that the L value in the skin of both males and females gradually decreased with increasing days of age. Yu et al. [6] found a correlation between a new SNP in the *ASIP* gene and black-bone chickens in Muchuan black-bone chickens, and the L value of white skin at the same age was higher than that of black skin. Li et al. [3] examined the luminosity index of the breast muscle of Xichuan black-boned chickens using a colorimeter test and found that the larger the “L”, the lower the degree of black color. Liu et al. [18] evaluated the skin color of the Dongxiang black-bone chicken breed from the age of 112 days after hatching and found that the skin color became lighter with the increase in chicken age. In this study, we investigated whether there was a significant difference in the degree of the black color of skin tissue between Tengchong Snow black meat chickens and white meat chickens at different ages. The results showed that at the same growth stage, the L value of the skin tissue in black meat chickens was significantly lower than that of the white meat chicken (*p* < 0.01), and the L value in the skin of both black and white meat chickens gradually increased with increasing age. The results of this study were consistent with those of Wang and Liu et al. [14,18]. This suggests that the number of melanocytes and the level of melanin deposition may gradually decrease with the chicken’s increasing age.

The degree of tissue blackness could mainly be determined by the content and distribution level of melanin, which is a high-protein molecule synthesized by melanosomes in melanocytes [19]. The results of this study showed that the content of the total melanin, eumelanin, and phaeomelanosome in different growth stages of Tengchong Snow black meat and white meat chickens were different, and the melanin content of the black meat chicken was higher than white meat chicken, though this difference was not significant. The content of melanin decreased continuously with increasing days of age, and the difference was not significant (*p* > 0.05), and this was negatively correlated with the L value in the skin tissues. Wang et al. [20] explored the effects of age, strain, feeding method, and sex on the melanin content in Taihe black-bone chickens and found that the proportion of total melanin decreased gradually with increasing days of age and, at a certain age, the total melanin was negatively correlated with the body weight of black-bone chickens, especially in 90-day-old silky-feathered black-bone chickens, where a higher body weight represented lower total melanin. Nishimura et al. [21] studied the relationship between the characteristics of melanocyte distribution and growth in the skeletal muscle of black-bone chickens and confirmed that the number of melanocytes in the muscle tissue was affected by age and found that, as the chickens aged, the number of melanocytes decreased, which could be related to the a-MSH hormone in keratin-forming cells. Han et al. [22] studied the distribution of melanocytes in 26 tissues or organs on the first day and at 2, 3, 4, 6, 10, and 23 weeks in Siyu black-bone chickens; the results showed that compared with Bailaihang chickens, Siyu black-bone chickens had a significant distribution of melanin in their skin, skeletal muscle (pectoral muscle and leg muscle), stomach muscle, and other visceral tissues, and the melanin deposition in Siyu black-bone chickens increased with their age. Jian et al. [12] determined the true and brown melanin content of Chishui black-bone chickens and Taihe black-bone chickens, and the results showed that the content of melanin (including true and brown melanin) in the leg muscles of Taihe black-bone chickens was significantly higher than that of Chishui black-bone chickens, but there was no significant difference in other tissues.

In recent years, with the rapid development of high-throughput sequencing technologies, it has become convenient to study the molecular mechanisms of phenotype formation in animals using genome-wide association studies (GWASs), transcriptomics, and single nucleotide polymorphisms (SNPs) [23,24]. Transcriptomic techniques have become convenient when studying the molecular mechanisms of phenotype formation in animals. Fan et al. [25] used transcriptomic analysis to study sheep skin from white- and black-coat animals and found that *DCT*, *MATP*, *TYR*, and *TYRP1* genes were the main functional genes for skin pigmentation in black sheep. Among them, the *TYRP1* gene was differentially expressed at the highest level in the skin tissue of black and white sheep. To determine the molecular mechanism of melanogenesis in muscle tissue, Yu et al. [26] used transcriptome sequencing technology to differentially characterize the transcriptome of the pectoral muscle of Muchuan black-bone chicken black meat and white meat muscle tissue, and *PMEL*, *RAB29*, and five solute carrier superfamily genes were identified as the possible major genes regulating melanin production in chicken muscle. Fan et al. [27] investigated the effect of *MC1R* 5′ sequence polymorphism on feather color in Taihang chickens and found a significant correlation between four SNPs and feather color. Therefore, in order to further investigate the functional genes affecting melanin biosynthesis on chicken skin, this study used transcriptome sequencing technology to mine the important genes affecting melanin deposition in the skin tissue of Tengchong Snow chickens. Among the obtained transcriptome sequencing reads, we screened a total of 44 differentially expressed genes, including 32 upregulated genes and 12 downregulated genes. The GO enrichment analysis results showed that the differentially expressed genes in relation to the skin’s melanin deposition and synthesis were mainly enriched in biological processes or cell components, such as organelles and cell membranes, including melanosomes, pigment granules, and pigment granule membranes. KEGG analysis showed that differentially expressed mRNAs in the skin tissue of black meat chickens were significantly enriched in melanogenesis, tyrosine metabolism, and RNA transport. We also identified new transcripts and new genes and suggested that these may play a key role in two different types of skin melanin deposition.

Many studies have shown that *TYR*, *MITF*, *DCT*, *MC1R*, *TYRP1*, *EDNRB2*, *TRPM1*, and *MLPH* genes are the main key functional genes affecting skin pigment deposition. *DCT*, *TYRP1*, and *TYR* genes are key factors in melanin synthesis, and these three melanogenic enzymes are metal-containing glycoproteins, have a single transmembrane α-helix, and share an approximately 40% amino acid sequence similarity. Tyrosinase (*TYR*) is a copper-containing enzyme that is widely found in plants and animals. It plays an important role in the synthesis of melanin and the involvement of water-soluble carrier family 45 member 2 (*SLC45A2*) in the intracellular processing and transport of TYR, which can have an impact on the expression and activity of TYR [28]. In addition, the rate and yield of melanin synthesis may be influenced by the expression and activity of *TYR* genes. It has been suggested that mutations in tyrosine (*TYR*) and tyrosinase-related enzyme 1 (*TYRP1*) may cause human oculocutaneous albinism type 1 (OCA1) and type 3 (OCA3) [29,30]. Differences in the plumage and skin color of chickens may be caused by the differential expression of the *TYR* gene. The retroviral insertion of the tyrosinase gene (*TYR*) has been associated with recessive white mutations in chickens [31]. Nam et al. [32] found, in a study on Korean native chickens, that specific mutations in the *TYR* gene can have important effects on melanosis in Korean native chickens. Yang et al. [33] identified three SNPs (C47G, T120C and T172C) on the chicken’s *TYR* gene, where loci C47G and T172C are missense mutations and loci T120C are synonymous mutations; association analysis showed that loci T120C were significantly associated with feather color in chickens. In this study, we found that the expression of the *TYR* gene was significantly higher in the skin tissue of Tengchong Snow black meat chickens than in white meat chickens. Additionally, all the expressions decreased gradually with an increase in the chickens’ age. This is in agreement with the findings of Khumpeerawat et al. [11]; the expression of the *TYR* gene was higher in black skin compared to light black and yellow-white skin chickens, and the expression of the gene decreased linearly with increasing age. In addition, Liu et al. [18] also evaluated the expression of the *TYR* gene in different day-old stages of skin color in Dongxiang black-bone chickens and found that the *TYR* gene expression was highest at the time of birth, which is consistent with the results of the present study. Ludwig et al. [34] found that the microphthalmia-related transcription factor (*MITF*) and sex-determining region Y-box 10 are involved due to their regulation of *DCT* gene expression and affect on melanin synthesis. In this study, we did not find significant differences in the expression of the *MITF* gene in the skin of black and white meat chickens. Our findings are in agreement with those of Dou et al. [8] and Zhang et al. [9]. Yuan et al. [35] studied the eyelids of Lindian chickens and also found that the expression of the *DCT* gene was higher in black eyelids than in yellow eyelids. In this study, the expression of the *DCT* gene was higher in the skin of all black meat chickens than in white meat chickens, and this expression response decreased gradually with increasing age. These results are consistent with the findings of Khumpeerawat et al. [11]. The expression of mRNAs in *TYR* and *DCT* genes was also found to be positively correlated with the skin’s total melanin, true melanin, and brown melanin content in black meat chickens in this study. The expression of the *TYR* gene was significantly correlated with brown melanin (*p* < 0.05), and the relative expression of the *DCT* gene was significantly correlated with total melanin and true melanin. Binstock et al. [36] reported that *DCT* is a key reactive enzyme that catalyzes the synthesis of true melanin, which converts dopaquinone to true melanin. The above studies reported with the results of the present study indicate that *TYR* and *DCT* genes are the major upregulated genes in the melanogenesis pathway. The level of their gene expression determines the pigmentation and synthesis of the animal organism, and a high level of expression contributes to the formation and deposition of melanin in the animal, resulting in color differences in the muscle and skin tissues.

EDNRB2, as an essential catalytic factor in the migration and differentiation of melanogenic fibroblasts, is upregulated in the early development and migration of melanogenic cells to the dorsolateral pathway [37]. In this study, we found that its expression was higher in the skin of black meat chickens than white meat chickens, and its expression also decreased gradually with increasing age. This differs from the findings of Kinoshita et al. [38], as their results showed that the *EDNRB2* gene was not significantly differentially expressed on the skin of adult black and non-black chickens. Wu et al. [39] compared the mRNA expression levels of the *EDNRB2* gene in the black and white feathers of the Shexiang duck and found that the gene was more significantly expressed in black feathers than white feathers, which is consistent with the results of the present study. Shi et al. [40] showed that the expression of the *EDNRB2* gene in muscle fibers decreased with age and that the melanin content also decreased with age, which is consistent with the results of the present study.

*TYRP1*, *MC1R*, and *GPR143* genes are thought to play an important role in the process of melanosis in chickens [41]. In this study, the results of RNA-seq analysis showed no significant difference in the expression of the above 3 known pigmentation genes in the skin tissue of 90-day-old black and white meat chickens of the Tengchong Snow chicken. To validate the results of RNA-seq analysis, we examined the expression of *TYRP1*, *MC1R*, and *GPR143* genes on the skin tissue of black and white meat chickens at 1, 42, and 90 days of age using qPCR and found that there was no significant difference in the expression of the three genes at 1, 42, and 90 days of age; *TYRP1* and *MC1R* genes at 42 days of age were the highest. Zhang et al. [42] found that *MC1R* and *TYRP1* genes were expressed at the highest level at 4 weeks of age in the skin tissues of black-bone chickens and then started to decrease. Yu et al. [26] found that *MC1R* and *TYRP1* were not differentially expressed in chicken muscle. However, Fan et al. [25] found that the *TYRP1* gene was differentially expressed at its highest level in black and white sheep skin, contrary to our results, suggesting that gene expression is differential in various animals. *GPR143* is a G protein-coupled receptor, and it has been found that the size, number, and maturation of melanosomes, and the level of the *MITF* gene, may influence the expression of melanin in the biosynthesis and deposition of the animal organism [43]. It has been reported that the *GPR143* gene plays an important role, mainly in the early stages of melanosome production [44]. In this study, the expression of the *GPR143* gene was higher at 1 day of age in black and white meat chickens than at 42 and 90 days of age. Liu et al. [45] studied black and white hair follicles in Bashu long-tailed chickens and found the greatest difference in the expression of the *GPR143* gene in black and white hair follicles, resulting in differences from the results of the present study. Chen et al. [46] found that the *GPR143* gene was differentially expressed between its highest and lowest values in the yellowness of skin through transcriptome sequencing, which suggests that differentially expressed *GPR143* genes may play an important role in the yellow pigmentation of chicken skin. Wu et al. [47] studied the expression pattern and localization of *GPR143* in sheep skin of different hair colors and found that the mRNA and protein levels of the *GPR143* gene were significantly higher in black sheep skin than in white sheep skin.

## 5. Conclusions

In conclusion, in this study, the skin color and melanin content of Tengchong Snow chickens were compared and evaluated at different days of age. The results showed that the L value of black meat chickens was lower than that of white meat chickens, and the melanin content was higher compared to that of white meat chickens. Additionally, the L value gradually increased with an increase in age, melanin content gradually decreased, and the L value was negatively correlated with the content of melanin. In addition, the transcriptomic profile of 90-day-old skin tissues of Tengchong Snow chickens was compared, and the results showed that the mRNA of the *TYR*, *DCT*, and *EDNRB2* genes was the highest in the skin tissue of Tengchong Snow chickens; this expression level was affected by their age, and the expression levels decreased with an increase in age. Our study preliminarily constructed an evaluation system for hedging the black quality traits of black-bone chickens and identified key candidate genes regulating melanin deposition, which could provide an important theoretical basis for the breeding of black-bone chickens.

## Figures and Tables

**Table 1:** Correlation analysis of skin L-value and melanin content.

	L Value	Total Melanin	Eumelanin	Phaeomelanosome
L value	1			
Total melanin	−0.987 **	1		
Eumelanin	−0.663 *	0.617 *	1	
Phaeomelanosome	−0.728 *	0.767 *	0.031	1

Note: “*” indicates a significant negative correlation (*p* < 0.05); “**” indicates a highly significant negative difference (*p* < 0.01).

## Data Availability

The data presented in this study are available on request from the corresponding author. The data are not publicly available due to privacy.

## Supplementary Materials

The following supporting information can be downloaded at: https://www.mdpi.com/article/10.3390/vetsci10050341/s1. Table S1: information regarding the specific primers used for the qPCR; Table S2: differential gene expression original data; Table S3: filtering of sample sequencing data; Table S4: comparison of reference area statistics; Table S5: statistical table of base information.
