# Supplementary material for: Transcriptome Profile Analysis Identifies Candidate Genes for the Melanin Pigmentation of Skin in Tengchong Snow Chickens
Source: Vet Sci. 2023 May 11;10(5):341. doi: 10.3390/vetsci10050341 (PMC10221249; doi:10.3390/vetsci10050341)
Supplement: Supplementary file 1 [file vetsci-10-00341-s001.zip › vetsci-2258038-supplementary/Table S1.docx]

**Table S1.** Information regarding the specific primers used for the qPCR.

| Gene | Primer sequence(5'-3') | Annealing temperature(℃) |
| --- | --- | --- |
| actin-β | F:GTGTGATGGTTGGTATGGGC  R:CTCTGTTGGCTTTGGGGTTC | 57.50 |
| *EDNRB2* | F: CGGTCCTCAGTCTTTGTG  R: CAGGTCTTGGTCCCAGTAG | 57.30 |
| *TYR* | F:TTGGAAGGCTTTGCTGAT  R: TTGGCTGCTGGGTAAACT | 58.00 |
| *DCT* | F: TCCCTCCTGTAACCAATG  R: AAGCAGCAGAACAAGCAC | 52.60 |
| *TYRP1* | F: CAGAAGCTCAGTTCCCTCG  R: TGGTTGAAGAAGCGTATGG | 57.30 |
| *GPR143* | F: GGACTGACATATGGCCCTCTG  R: AATAACCACCAGAAGCCAGCA | 59.50 |
| *MC1R* | F: TCCGTCGTGTCCTCCCTCT  R: CCAGCGCGAACATGTGAA | 59 |
| *MITF* | F: TGTGACTGAACCAACTGGCACTTAC  R: TGCTCCGCCTGCTACTCGTT | 57.50 |
