# Supplementary material for: Transcriptome Profile Analysis Identifies Candidate Genes for the Melanin Pigmentation of Skin in Tengchong Snow Chickens
Source: Vet Sci. 2023 May 11;10(5):341. doi: 10.3390/vetsci10050341 (PMC10221249; doi:10.3390/vetsci10050341)
Supplement: Supplementary file 1 [file vetsci-10-00341-s001.zip › vetsci-2258038-supplementary/Table S3.docx]

**Table S3.** Filtering of sample sequencing data.

| Sample | Raw Data | Clean Data(%) | N (%) |
| --- | --- | --- | --- |
| Bc-1 | 53560184 | 53299200 (99.51%) | 0 (0.00%) |
| Bc-2 | 49669728 | 49440548 (99.54%) | 0 (0.00%) |
| Bc-3 | 46740606 | 46500814 (99.49%) | 0 (0.00%) |
| Bc-4 | 38975920 | 38768854 (99.47%) | 0 (0.00%) |
| Bc-5 | 60077536 | 59809112 (99.55%) | 0 (0.00%) |
| Bc-6 | 66239134 | 65955262 (99.57%) | 0 (0.00%) |
| Wc-1 | 74461034 | 74102524 (99.52%) | 0 (0.00%) |
| Wc-2 | 54909370 | 54603034 (99.44%) | 0 (0.00%) |
| Wc-3 | 47683832 | 47466318 (99.54%) | 0 (0.00%) |
| Wc-4 | 48374394 | 48119016 (99.47%) | 0 (0.00%) |
| Wc-5 | 48523540 | 48266618 (99.47%) | 0 (0.00%) |
| Wc-6 | 47462072 | 47190740 (99.43%) | 0 (0.00%) |

Note: Bc for the black meat skin, and Wc for the white meat skin. The following is below.
