# Supplementary material for: Transcriptome Profile Analysis Identifies Candidate Genes for the Melanin Pigmentation of Skin in Tengchong Snow Chickens
Source: Vet Sci. 2023 May 11;10(5):341. doi: 10.3390/vetsci10050341 (PMC10221249; doi:10.3390/vetsci10050341)
Supplement: Supplementary file 1 [file vetsci-10-00341-s001.zip › vetsci-2258038-supplementary/Table S4.docx]

**Table S4.** Comparison of reference area statistics.

| Sample | exon | Intron | intergenic |  | |
| --- | --- | --- | --- | --- | --- |
| Bc-1 | 72.71 | 11.59 | 15.70 | |  |
| Bc-2 | 72.79 | 11.87 | 15.34 | |  |
| Bc-3 | 73.35 | 12.36 | 14.28 | |  |
| Bc-4 | 74.58 | 10.46 | 14.96 | |  |
| Bc-5 | 75.11 | 11.36 | 13.53 | |  |
| Bc-6 | 73.18 | 12.22 | 14.60 | |  |
| Wc-1 | 69.27 | 13.44 | 17.29 | |  |
| Wc-2 | 72.60 | 11.40 | 16.00 | |  |
| Wc-3 | 72.97 | 12.08 | 14.95 | |  |
| Wc-4 | 75.39 | 10.16 | 14.45 | |  |
| Wc-5 | 73.06 | 11.84 | 15.09 | |  |
| Wc-6 | 73.14 | 10.10 | 16.77 | |  |
