# Supplementary material for: Transcriptome Profile Analysis Identifies Candidate Genes for the Melanin Pigmentation of Skin in Tengchong Snow Chickens
Source: Vet Sci. 2023 May 11;10(5):341. doi: 10.3390/vetsci10050341 (PMC10221249; doi:10.3390/vetsci10050341)
Supplement: Supplementary file 1 [file vetsci-10-00341-s001.zip › vetsci-2258038-supplementary/Table S5.docx]

**Table S5.** Statistical table of base information.

| Sample | RawData  (bp) | | BF_Q20  (%) | BF_Q30  (%) | BF_N  (%) | BF_GC  (%) | CleanData  (bp) | AF_Q20  (%) | AF_Q30  (%) | AF_N  (%) | AF_GC  (%) |
| --- | --- | --- | --- | --- | --- | --- | --- | --- | --- | --- | --- |
| Bc-1 | 8034027600 | | 97.19 | 92.71 | 0.00 | 47.92 | 7940852448 | 97.41 | 92.99 | 0.00 | 47.80 |
| Bc-2 | 7450459200 | 97.23 | | 92.71 | 0.00 | 47.39 | 7374441127 | 97.43 | 92.97 | 0.00 | 47.28 |
| Bc-3 | 7011090900 | 97.27 | | 92.89 | 0.00 | 48.03 | 6930466821 | 97.50 | 93.19 | 0.00 | 47.91 |
| Bc-4 | 5846388000 | 97.05 | | 92.41 | 0.00 | 48.22 | 5780290940 | 97.29 | 92.71 | 0.00 | 48.10 |
| Bc-5 | 9011630400 | 97.27 | | 92.85 | 0.00 | 47.77 | 8914467219 | 97.48 | 93.11 | 0.00 | 47.67 |
| Bc-6 | 9935870100 | 97.12 | | 92.48 | 0.00 | 47.31 | 9831014193 | 97.31 | 92.73 | 0.00 | 47.20 |
| Wc-1 | 11169155100 | 97.15 | | 92.55 | 0.00 | 46.83 | 11037453287 | 97.36 | 92.82 | 0.00 | 46.71 |
| Wc-2 | 8236405500 | 97.07 | | 92.42 | 0.00 | 47.59 | 8128134393 | 97.32 | 92.74 | 0.00 | 47.47 |
| Wc-3 | 7152574800 | 97.28 | | 92.79 | 0.00 | 47.31 | 7070918896 | 97.49 | 93.06 | 0.00 | 47.20 |
| Wc-4 | 7256159100 | 97.00 | | 92.30 | 0.00 | 47.73 | 7163746086 | 97.25 | 92.61 | 0.00 | 47.63 |
| Wc-5 | 7278531000 | 97.05 | | 92.42 | 0.00 | 47.81 | 7193669665 | 97.29 | 92.72 | 0.00 | 47.70 |
| Wc-6 | 7119310800 | 97.01 | | 92.38 | 0.00 | 47.67 | 7028195722 | 97.27 | 92.71 | 0.00 | 47.54 |
